# Supplementary material for: Androgen deprivation–mediated activation of AKT is enhanced in prostate cancer with TMPRSS2:ERG fusion
Source: J Clin Invest. 2025 Oct 2;135(23):e192368. doi: 10.1172/JCI192368 (PMC12646650; doi:10.1172/JCI192368)
Supplement: Supplemental data [file jci-135-192368-s324.pdf]

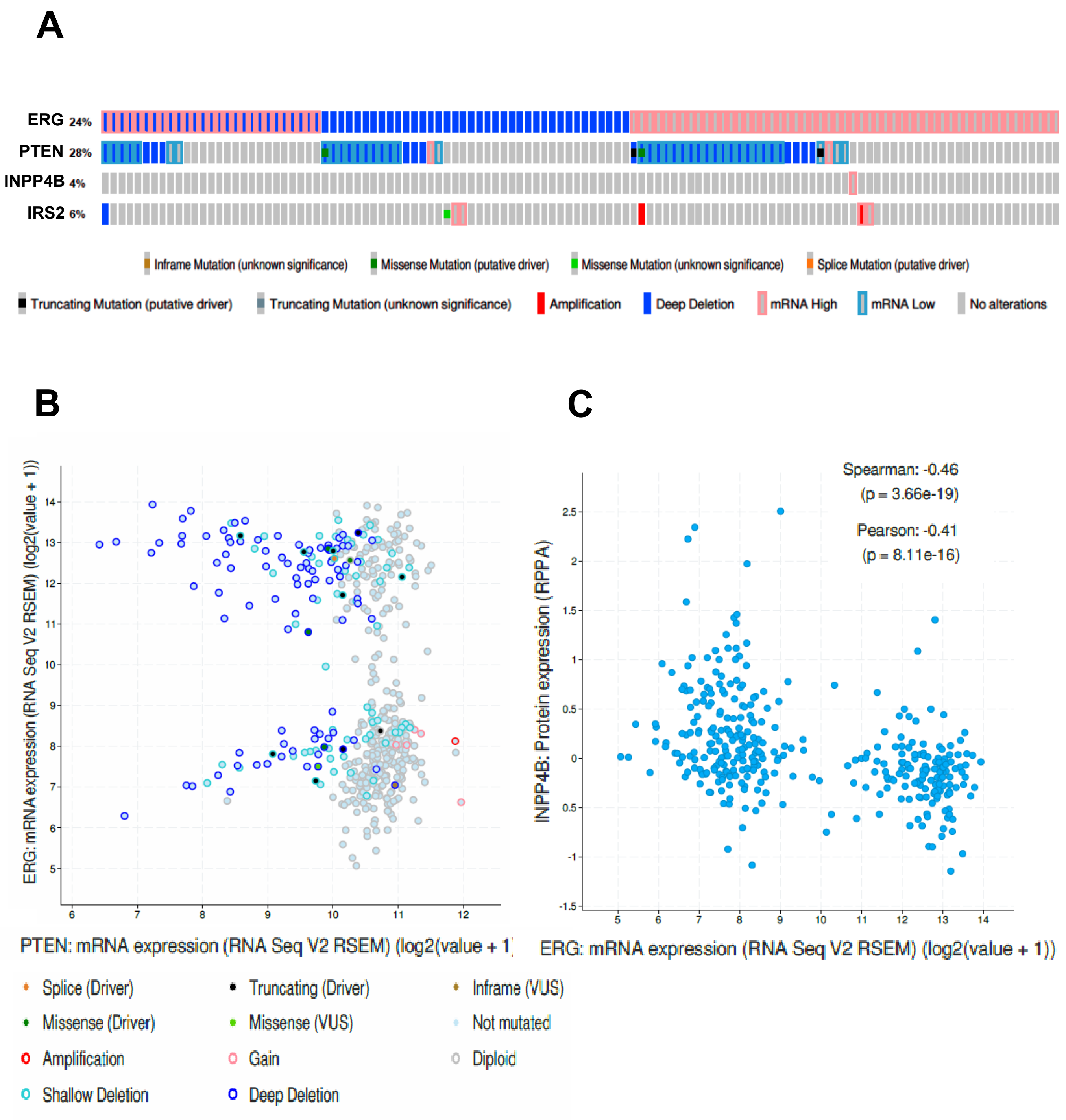

Figure S1. Associations between *T:E* fusion and PTEN in primary PCa. (A) Oncoprint showing alterations in *PTEN*, *INPP4B*, and *IRS2* in *T:E* fusion positive tumors in TCGA. Note that a subset of the *T:E* fusion positive tumors are scored as deep deletions, which likely reflects the fusion being mediated by interstitial deletion. **(B)** Correlation between ERG and PTEN mRNA in TCGA primary PCa showing PTEN mRNA levels in *PTEN* intact tumors are not decreased in *T:E* fusion tumors. **(C)** Correlation between ERG mRNA and INPP4B protein in TCGA primary PCa.

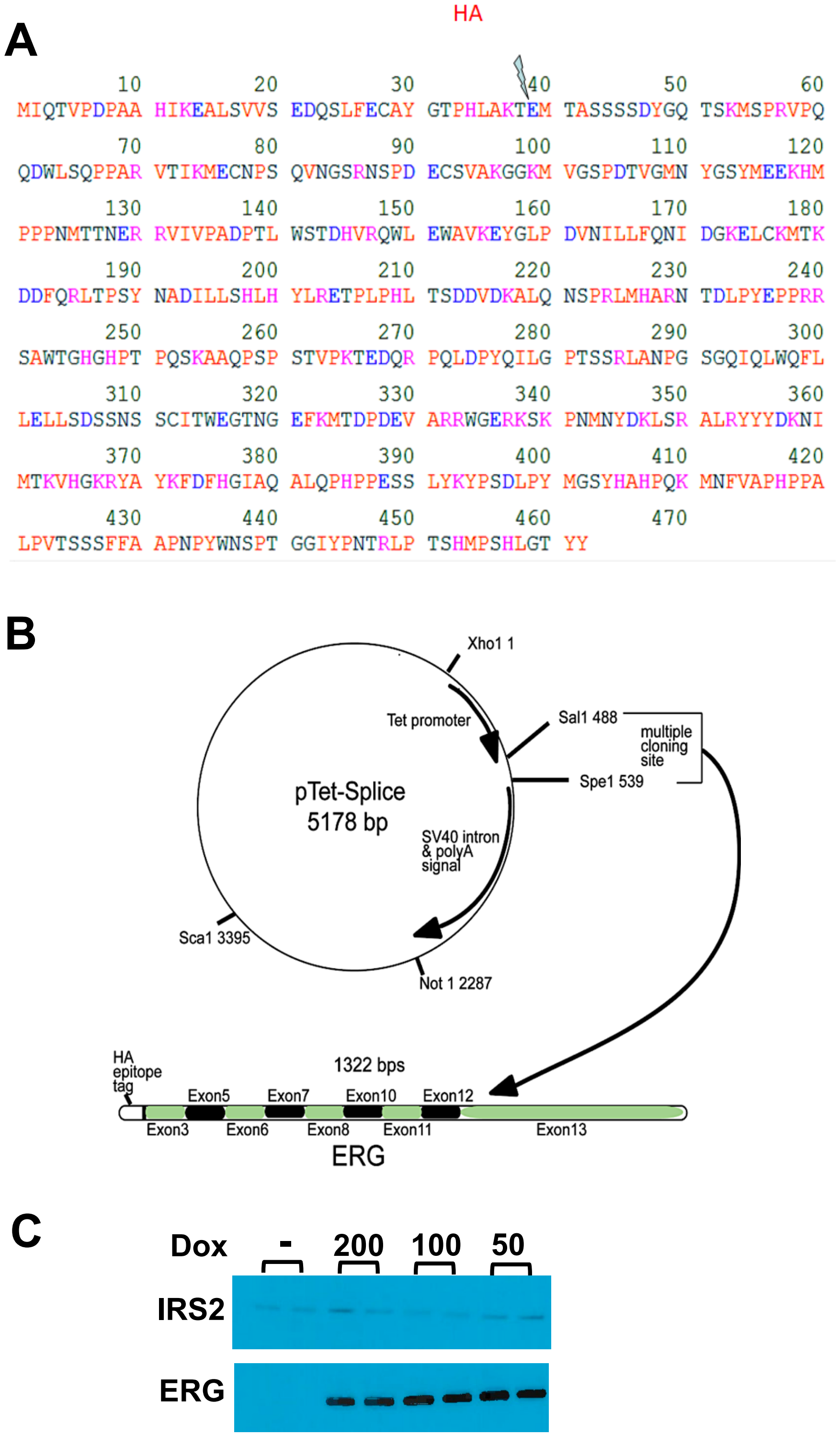

Figure S2. Construction of pTET-ERG expression vector. (A) HA epitope tag was inserted into human ERG with deletion of amino acids 1-39. **(B)** The HA-tagged ERG was cloned into the pTET-Splice plasmid to yield pTET-ERG. **(C)** LNCaP cells stably expressing the DOX-inducible HA-tagged ERG were treated with DOX (0 -200 ng/ml) for 2 days and assessed by immunoblotting for IRS2 and ERG.

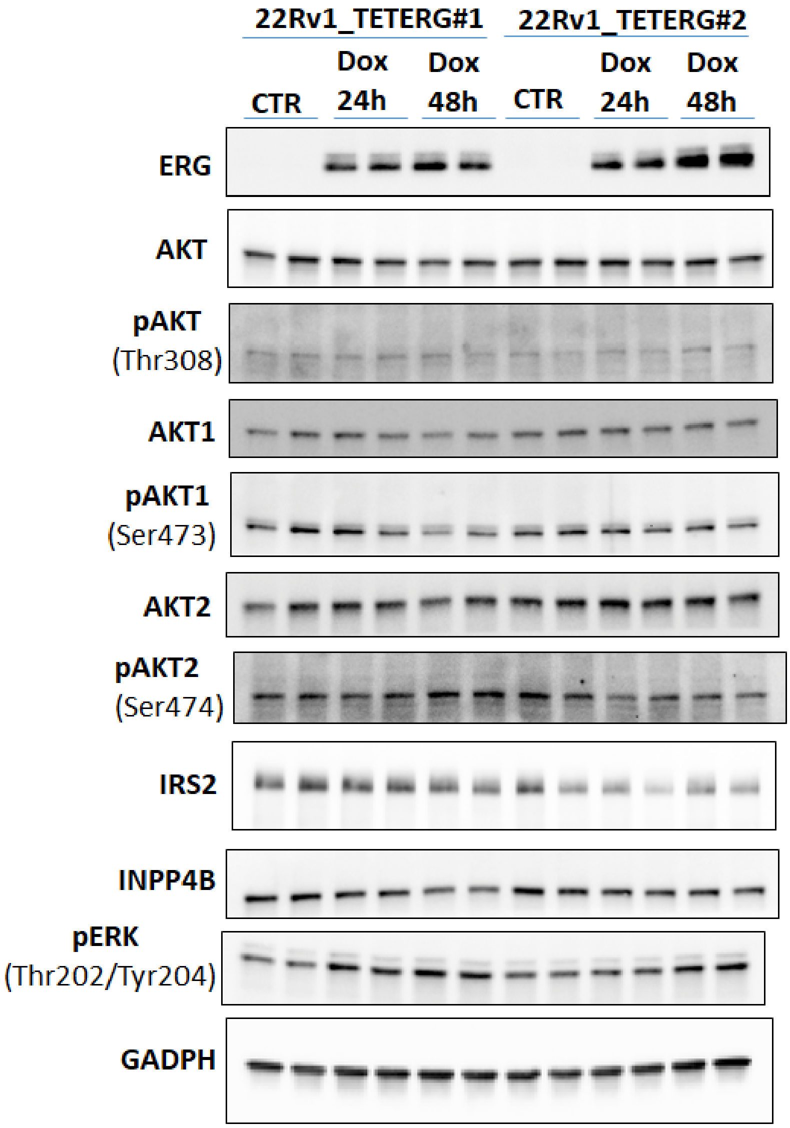

Figure S3. Effects of acute ERG induction in 22Rv1 cells. Two independent 22Rv1 lines with Dox-inducible expression of ERG were treated with Dox (50 ng/ml) versus vehicle for 48 hours followed by immunoblotting as indicated. Biological replicates are shown for each condition.

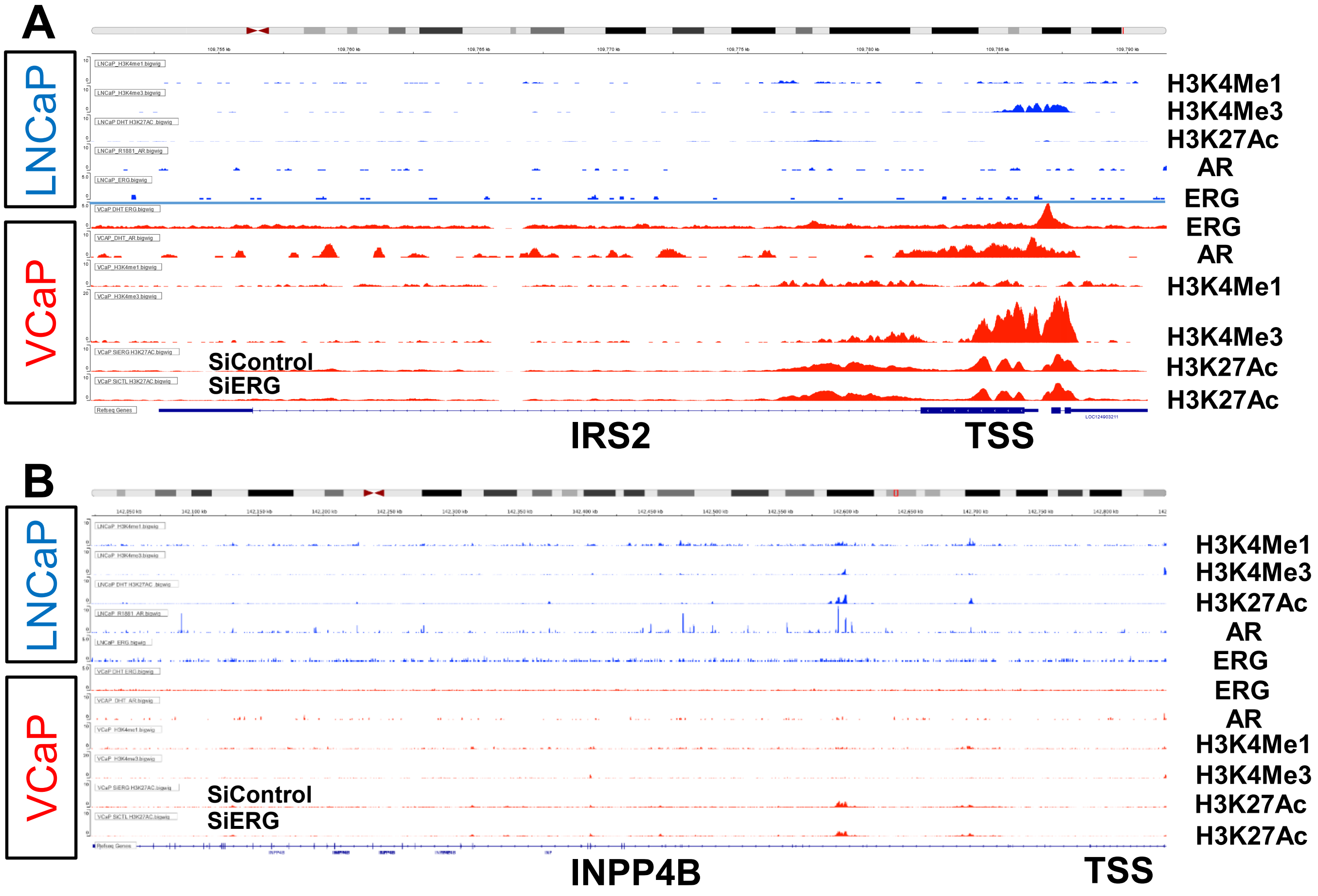

Figure S4. Binding profile of AR and ERG to the IRS2 and INPP4B transcription start sites in VCaP (*T:E* fusion positive) and LNCaP (*T:E* fusion negative) PCa cells. (A) ChIP-seq profiles for ERG, AR, and histone modifications H3K27Ac, H3K4Me3, and H3K4Me1 across a segment of genomic DNA encompassing *IRS2*. Each row represents a different ChIP-seq experiment with peaks indicating regions of protein-DNA interaction or histone modification enrichment. All data were retrieved from GEO, with accession number listed in Methods. Data ranges were set as 0-5 for ERG track, 0-20 for H3K4Me3 VCaP track, and 0-10 for all other tracks. **(B)** Binding profiles at *INPP4B* gene. Data ranges were set as 0-5 for ERG track, and 0-10 for all other tracks.

A

NAME	NES	NOM p-val	FDR q-val
HALLMARK_ANDROGEN_RESPONSE	1.4826885	0.046348315	1
HALLMARK_FATTY_ACID_METABOLISM	1.3813833	0.11127596	0.8967761
HALLMARK_PI3K_AKT_MTOR_SIGNALING	1.2420679	0.22136223	1
HALLMARK_UV_RESPONSE_DN	0.9845556	0.48351648	1
HALLMARK_P53_PATHWAY	0.9074609	0.58640224	1
HALLMARK_MYC_TARGETS_V1	-2.2282867	0	0.003571428
HALLMARK_E2F_TARGETS	-2.1276696	0	0.004306548
HALLMARK_MYC_TARGETS_V2	-1.8752728	0.006329114	0.02614438
HALLMARK_INTERFERON_GAMMA_RESPONSE	-1.7219411	0.029605264	0.07473872
HALLMARK_TNFA_SIGNALING_VIA_NFKB	-1.6377237	0.013888889	0.11279604
HALLMARK_COAGULATION	-1.6106604	0.044692736	0.11113012
HALLMARK_COMPLEMENT	-1.5046288	0.08522727	0.17373814
HALLMARK_INFLAMMATORY_RESPONSE	-1.4553362	0.08064516	0.19356337
HALLMARK_ESTROGEN_RESPONSE_LATE	-1.4501067	0.05204461	0.1760719
HALLMARK_DNA_REPAIR	-1.4206593	0.084269665	0.18824485

B

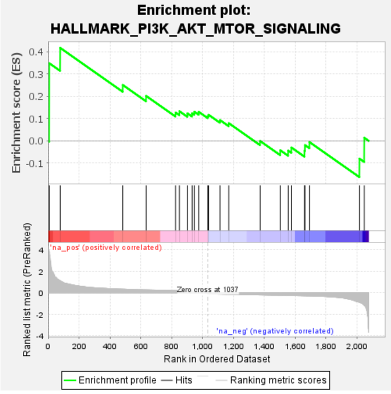

Normalized Enrichment Score (NES) 1.24
Nominal p-value 0.221
FDR q-value 1.0

C

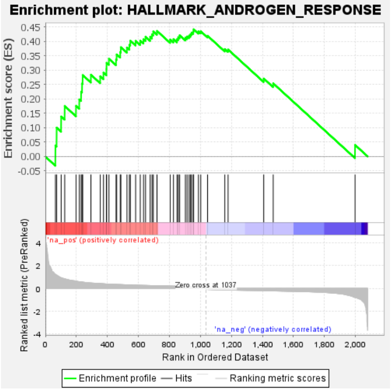

Normalized Enrichment Score (NES) 1.48
Nominal p-value 0.046
FDR q-value 1.0

D

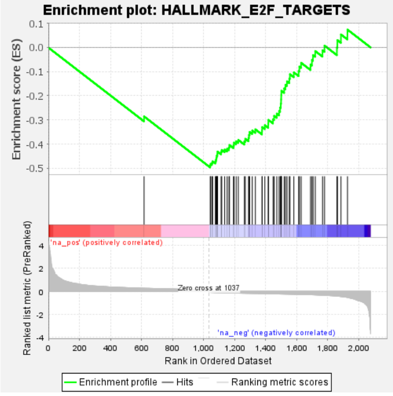

Normalized Enrichment Score (NES) -2.13
Nominal p-value 0.0
FDR q-value 0.004

Figure S5. Gene set enrichments in siERG versus control VCaP cells. (A) Gene sets most highly increased and decreased in VCaP cells treated with nontarget control siRNA versus ERG targeted siRNA. Gene sets were determined using the GSEA browser (www.gsea-msigdb.org) version 4.4.3 and a rank list with genes that were significantly altered (p <.05) is shown. **(B-D)** Enrichment for Hallmark PI3K AKT MTOR (B), Androgen Response (C), and E2F Targets (D) gene sets.

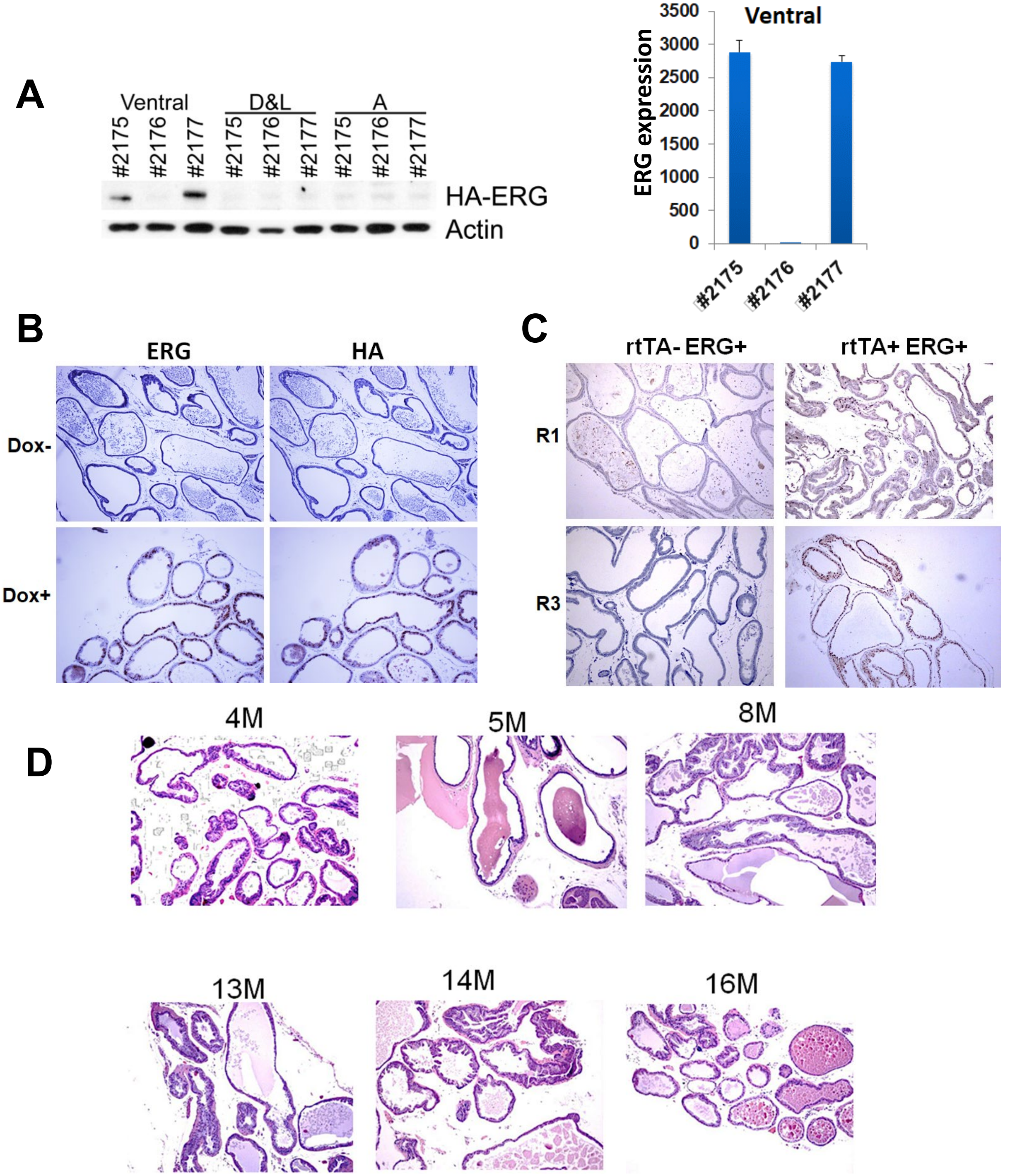

Figure S6. Doxycycline induction of ERG in mouse prostate. (A) Mice expressing probasin-rtTA and tet0-HA-ERG were fed doxycycline chow for ~4 weeks and then sacrificed. Prostates were dissected into ventral, dorsolateral (D&L), and anterior (A) lobes and assessed for ERG by immunoblotting (left). Ventral lobe was further assessed by qRT-PCR for human ERG, normalized to untreated prostate (right). **(B)** ERG and HA expression in ventral prostate after 24 days of DOX or control chow. **(C)** Two independent lines of tetO-ERG mice (R1 and R3) were bred to coexpress the probasin-rtTA transgene (rtTA+ ERG+) in comparison with those not coexpressing probasin-rtTA (rtTA- ERG+). Mice were fed DOX chow and sacrificed to assess ERG induction, which confirmed the ERG expression was probasin-rtTA dependent. **(D)** Representative images of prostates from probasin-rtTA; tetO-ERG mice fed DOX chow for 4 – 16 months.

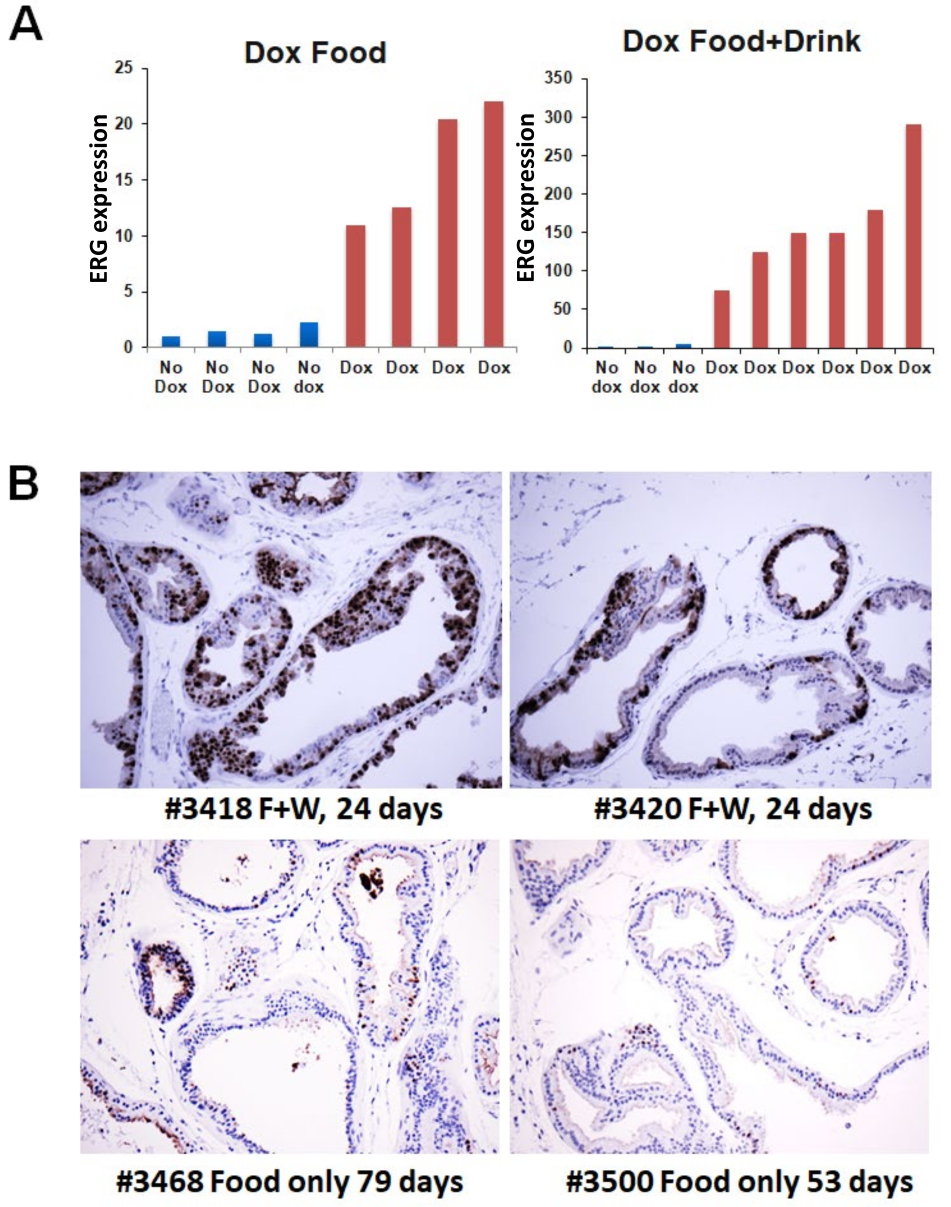

Figure S7. ERG expression is increased by adding doxycycline to water in addition to chow. Probasin-rtTA;tetO-ERG;*Pten*$^{+/-}$ mice were treated with DOX in chow or in chow plus water (or no DOX) for ~1-3 months. ERG expression in ventral prostate was then assessed by qRT-PCR (normalized to no Dox) **(A)** or IHC **(B)**.

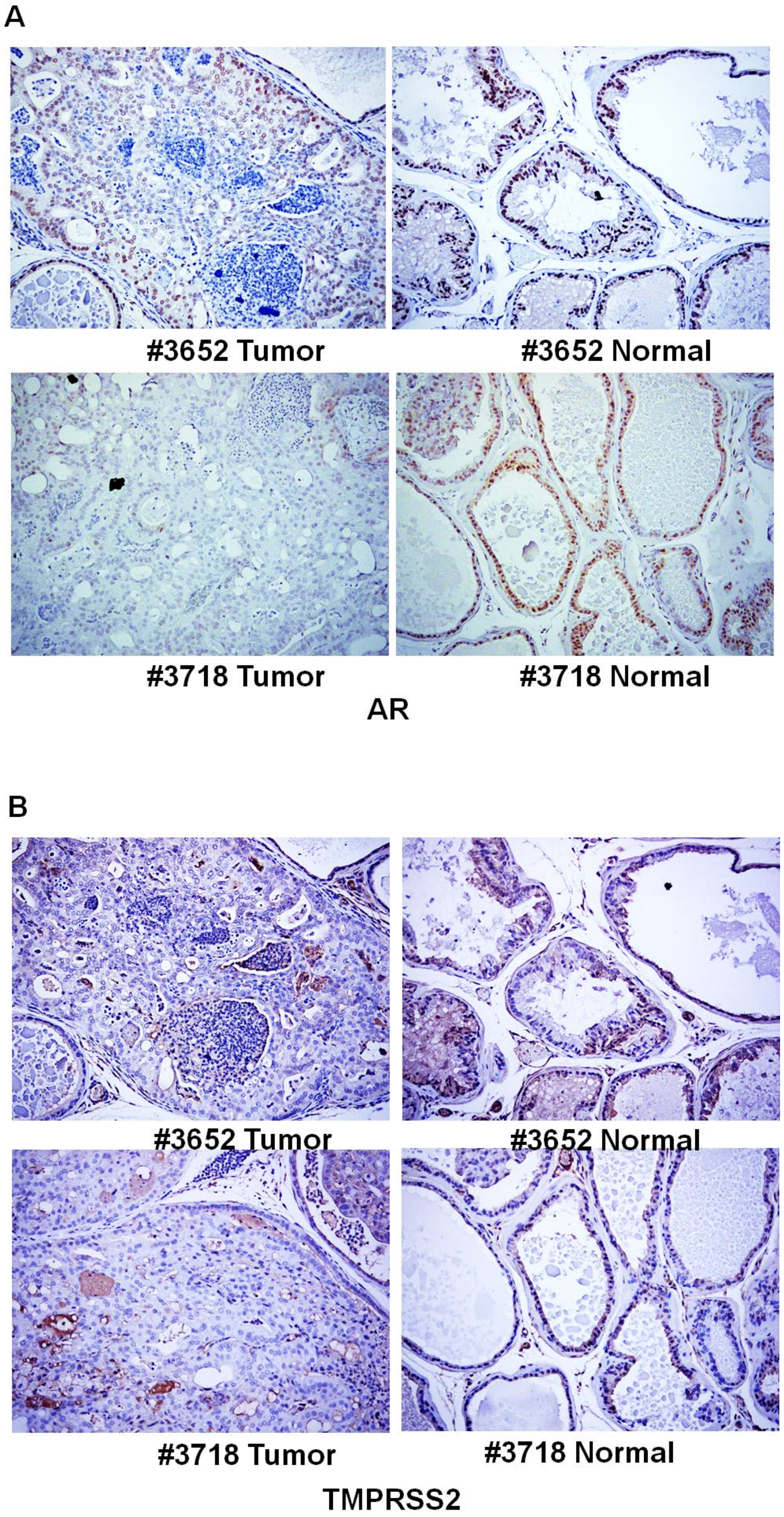

Figure S8. Decreased expression of AR and TMPRSS2 in tumor foci. (A) Probasin-rtTA;tetO-ERG;*Pten*$^{+/-}$mice treated with DOX (food and water) for ~12 months were sacrificed. Areas showing tumor and normal appearing areas were identified by H&E and then assessed by IHC for AR. **(B)** Additional sections from blocks in (A) were stained for TMPRSS2.

A

Gene Set	NES	FDR q-val
HALLMARK_MYOGENESIS	4.5403647	0
HALLMARK_EPITHELIAL_MESENCHYMAL_TRANSITION	2.2551758	0.18933873
HALLMARK_COAGULATION	1.91231	0.18244165
HALLMARK_KRAS_SIGNALING_DN	1.7335945	0.20586582
HALLMARK_XENOBIOTIC_METABOLISM	1.7249898	0.1690551
HALLMARK_MYC_TARGETS_V1	-7.6978273	0
HALLMARK_UNFOLDED_PROTEIN_RESPONSE	-4.7922244	0
HALLMARK_OXIDATIVE_PHOSPHORYLATION	-4.0124297	0
HALLMARK_PROTEIN_SECRETION	-3.3994312	0
HALLMARK_MTORC1_SIGNALING	-3.3154247	0
HALLMARK_MYC_TARGETS_V2	-3.0720851	0
HALLMARK_DNA_REPAIR	-2.9756088	0
HALLMARK_PI3K_AKT_MTOR_SIGNALING	-2.6895525	0
HALLMARK_MITOTIC_SPINDLE	-2.2741077	7.52E-04
HALLMARK_G2M_CHECKPOINT	-2.2648387	6.77E-04
HALLMARK_E2F_TARGETS	-2.128129	0.001034965
HALLMARK_FATTY_ACID_METABOLISM	-1.5648015	0.054587305
HALLMARK_GLYCOLYSIS	-1.4887736	0.072746694
HALLMARK_HEME_METABOLISM	-1.4637861	0.07127577
HALLMARK_TGF_BETA_SIGNALING	-1.2542614	0.19703972
HALLMARK_P53_PATHWAY	-1.2404442	0.19755296

B

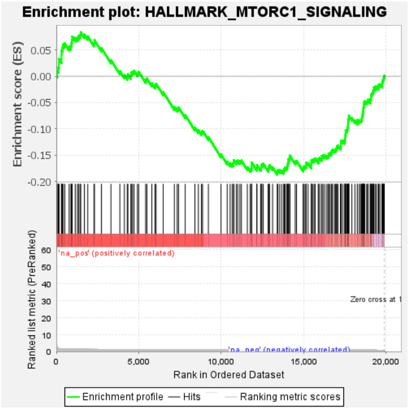

Normalized Enrichment Score (NES) -3.32
Nominal p-value 0.0
FDR q-value 0.0

C

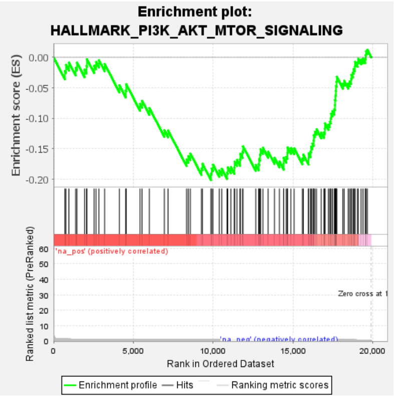

Normalized Enrichment Score (NES) -2.69
Nominal p-value 0.0
FDR q-value 0.0

D

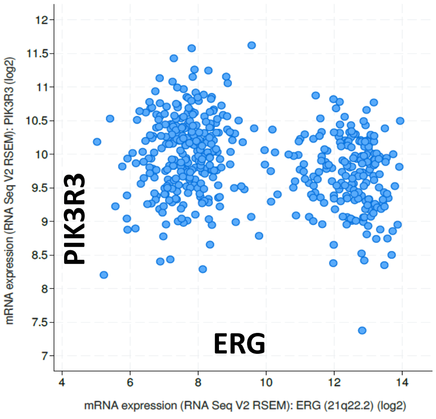

Spearman: -0.21 (p = 3.112e-6)   Pearson: -0.24 (p = 5.82e-8)

Figure S9. Hallmark gene set enrichments in response to initiating doxycycline in probasin-rtTA;tetO-ERG;*Pten*$^{+/-}$ mice. (A) Ventral prostate was isolated from mice treated for 3-6 days with DOX (food and drinking water) or control. RNA was extracted and analyzed on Affymetrix microarrays, followed by GSEA. Hallmark gene sets altered (increased or decreased) with FDR <0.25 are shown. **(B)** Enrichment for MTORC1 Signaling in response to DOX induction. **(C)** Enrichment for PI3K AKT MTOR Signaling in response to DOX induction. **(D)** Correlation between *T:E* fusion and PIK3R3 in TCGA primary PCa data set.

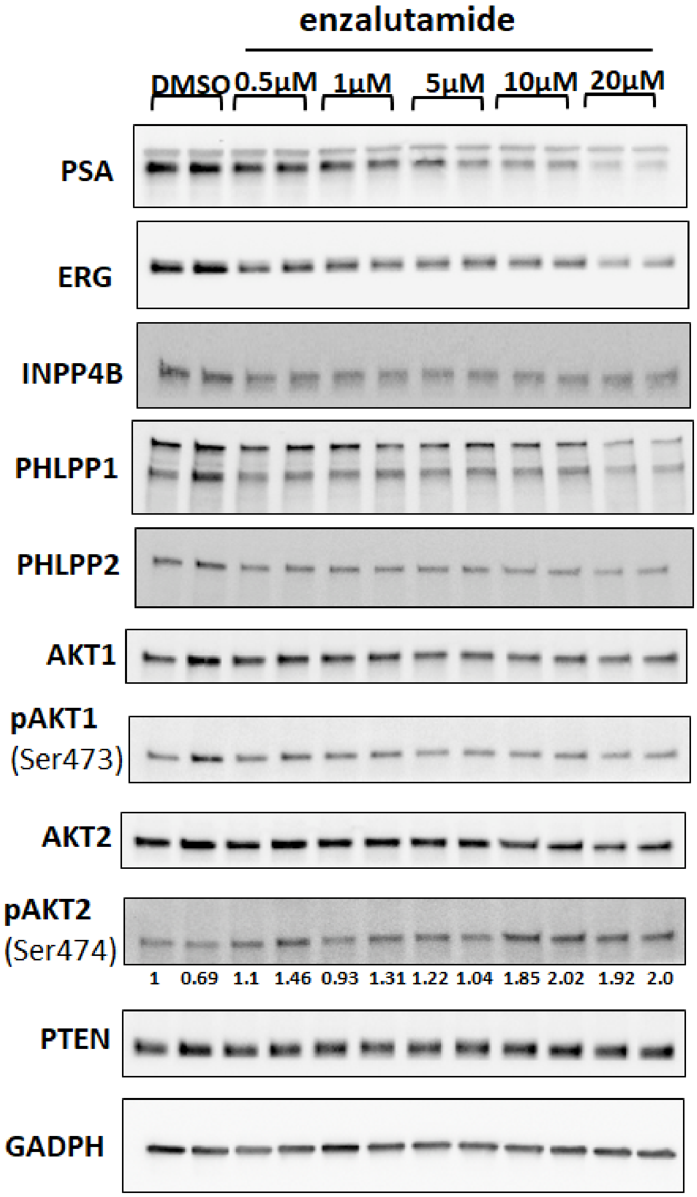

Figure S10. Effects of AR inhibition of ERG and AKT activation in *T:E* fusion cells. VCaP cells were treated for 48 hours with enzalutamide (0 – 20 µM) followed by immunoblotting as indicated. Biological replicates are shown for each condition. Band intensities for pAKT2 were quantified and values shown are normalized to the level in the first control.

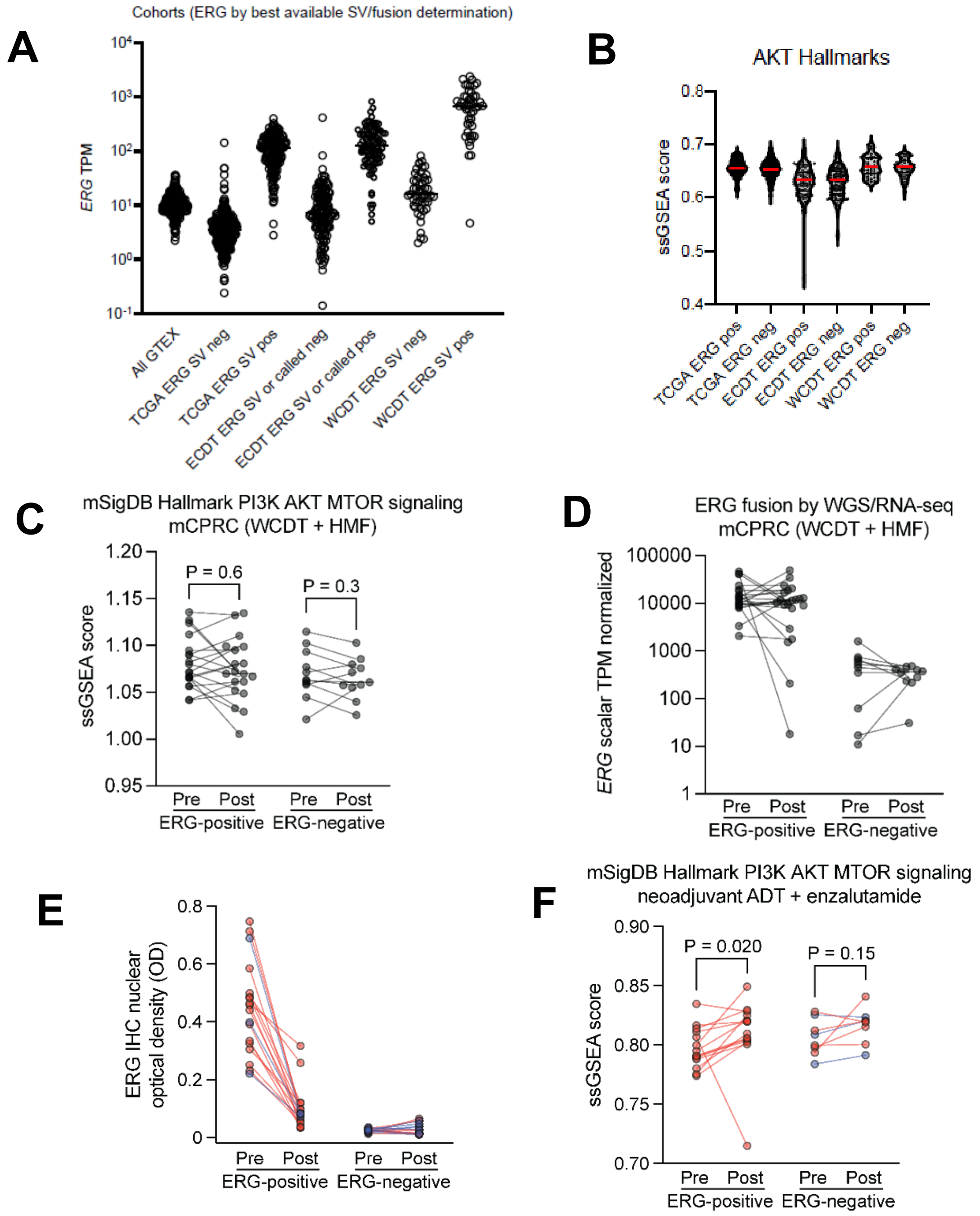

Figure S11. AKT activity is not increased in *T:E* positive CRPC. (A) Samples were separated into *T:E* fusion positive versus negative tumors based on genomic determination of structural variation/fusion status and each group was then assessed for ERG expression (transcripts per million, TPM) based on RNA-seq data. Samples are nonneoplastic prostate (GTEX, n= 243), TCGA primary PCa (n = 500), East Coast Dream Team CRPC (ECDT, n = 236), West Coast Dream Team (WCDT, n = 99). (B) ssGSEA showing enrichment for the Hallmark PI3K_AKT_MTOR gene set in each tumor. (**C, D**) Hallmark PI3K_AKT_MTOR gene set enrichment (**C**) and ERG expression (**D**) in matched CRPC tumors before and after AR-targeted therapy. (**E, F**) Tumors from figure 6 that are PTEN intact are shown in blue. The ssGSEA score in *PTEN* intact *T:E* tumors and some *T:E* negative tumors could not be determined due to low levels of residual tumor for which RNA-seq could not be done.

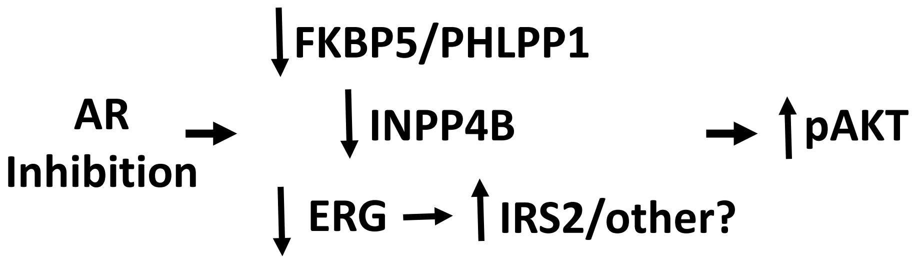

Figure S12. Potential mechanisms driving AKT activation in response to AR inhibition in *T:E* fusion tumors. AR inhibition decreases expression of FKBP5, which may act as a scaffold for PHLPP1-mediated dephosphorylation of AKT, leading to increased pAKT. AR inhibition also decreases INPP4B, leading to increased levels of $PI(3,4)P_2$ that can mediate AKT recruitment and activation. AR inhibition in *T:E* fusion tumors also decreases ERG, which can increase IRS2 and thereby enhance signaling by receptor tyrosine kinases and downstream activation of PI3K/AKT, and potentially by other pathways. The decrease in ERG may also act through other mechanisms that remain to be determined. Notably, the decrease in INPP4B may further amplify this increase in receptor tyrosine kinase signaling.

id	baseMean	log2FoldChange	lfcSE	pvalue	padj
HEPACAM2	172.2189742	2.87	0.174380802	6.72378E-61	1.66454E-57
FAM135B	22.40023764	1.97	0.449759386	1.23941E-05	0.000566102
LRRTM4	15.89257932	1.93	0.49895549	0.000109134	0.003377167
TLE4	21.06514634	1.93	0.438545253	1.13006E-05	0.000521938
NOV	100.46026	1.77	0.208452068	1.772E-17	5.22234E-15
NTN4	157.2038502	1.66	0.163305908	3.07944E-24	1.58822E-21
FFAR4	22.70872809	1.65	0.414658981	6.82838E-05	0.002265996
FLG	14.95139564	1.64	0.505036379	0.001204838	0.023861585
LAMA1	14.41171099	1.50	0.499025754	0.002624341	0.042023399
LBH	15.47188767	1.47	0.484838533	0.002509955	0.040453407
WNT5A	127.6402137	1.45	0.176076246	1.81439E-16	4.67885E-14
CACNA1G	48.47064561	1.45	0.282732374	3.08297E-07	2.10834E-05
TSC22D3	25.45945533	1.45	0.383975393	0.000166223	0.004727664
C7orf63	194.2586594	1.41	0.163289388	5.42856E-18	1.72294E-15
NEFH	95.16640014	1.37	0.211662919	1.06862E-10	1.4223E-08
IRS2	1998.574656	1.32	0.060898251	1.4374E-103	5.9308E-100
GALNT12	18.39030018	1.30	0.438073216	0.003041958	0.046890852
ATOH1	69.51044815	1.28	0.235081597	5.3179E-08	4.47789E-06
SLC45A3	5064.284271	1.26	0.053552033	3.6754E-122	2.2747E-118
HERC5	31.47328552	1.25	0.341728989	0.000258094	0.00678278
ADAMTS1	493.5041804	1.24	0.10137624	2.26095E-34	2.79861E-31
CRISPLD1	51.6313331	1.22	0.26924366	6.12163E-06	0.000306775
PCDH19	39.693299	1.18	0.308107708	0.00013012	0.003862408
TMPRSS2	3112.915769	1.16	0.046078015	2.9963E-140	3.7088E-136
CACNG4	73.69423266	1.13	0.221259184	3.54999E-07	2.40119E-05
ADCY2	78.01599669	1.12	0.215496176	1.97028E-07	1.4262E-05
HSPA12B	30.20897062	1.12	0.353669443	0.001603603	0.029848717
SPTSSB	104.1757232	1.11	0.183590599	1.72841E-09	1.99947E-07
MME	85.18886449	1.08	0.206649147	1.70962E-07	1.2748E-05
SPOCK3	262.7780904	1.08	0.117246231	3.77913E-20	1.41752E-17
MAFB	414.7640062	1.05	0.09699994	4.42181E-27	2.60634E-24
CPAMD8	514.0265997	1.04	0.092350749	1.655E-29	1.07819E-26
FAM84A	628.3461539	1.02	0.089947876	9.20847E-30	6.33236E-27
FAM43A	730.9535563	1.00	0.084407278	2.84302E-32	2.34606E-29

Table S1. Genes with increased expression in ERG versus nontarget control siRNA treated VCaP cells. Genes increased at least 2-fold with adjusted p value <.05 are shown.
