# Supplementary material for: Androgen deprivation–mediated activation of AKT is enhanced in prostate cancer with TMPRSS2:ERG fusion
Source: J Clin Invest. 2025 Oct 2;135(23):e192368. doi: 10.1172/JCI192368 (PMC12646650; doi:10.1172/JCI192368)
Supplement: Unedited blot and gel images [file jci-135-192368-s325.pdf]

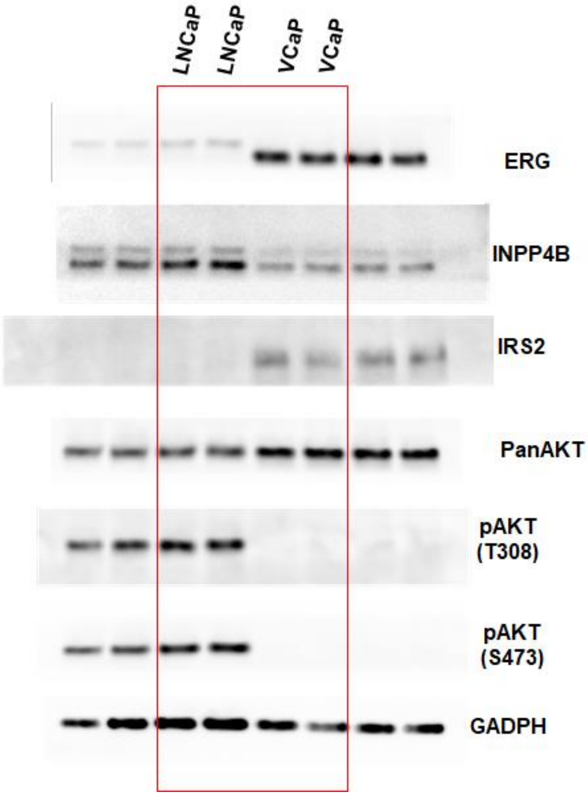

Uncropped gels corresponding to Figure 1F

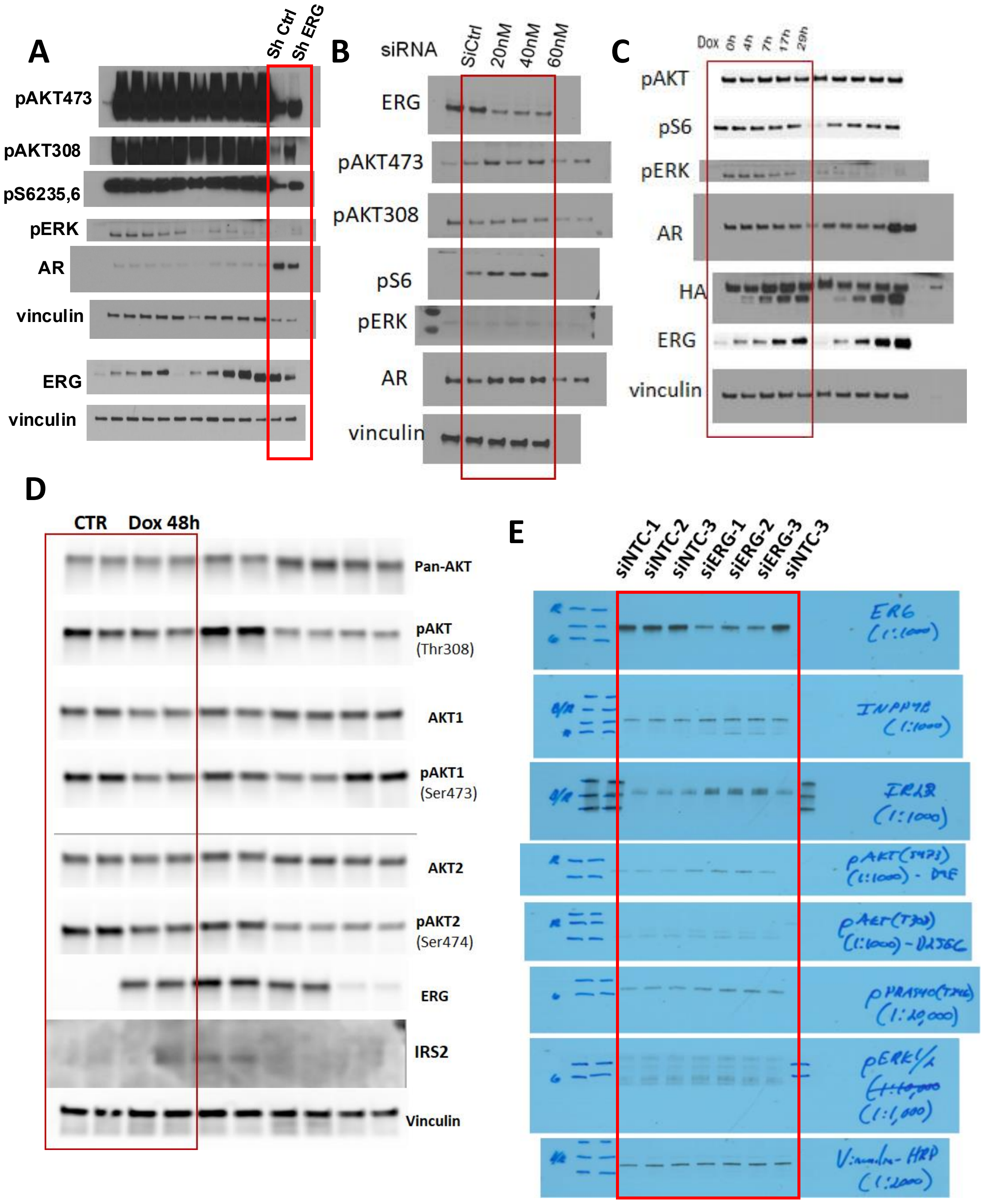

Uncropped gels corresponding to Figure 2. In Figure 2A, blots for the shCtrl and shERG were from the same lysate but ERG was run on a second gel, and the vinculin control for it is shown below the ERG blot.

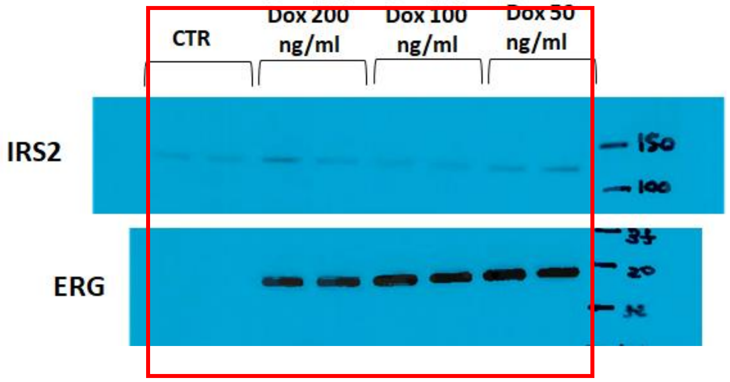

Uncropped gels corresponding to Figure S2C

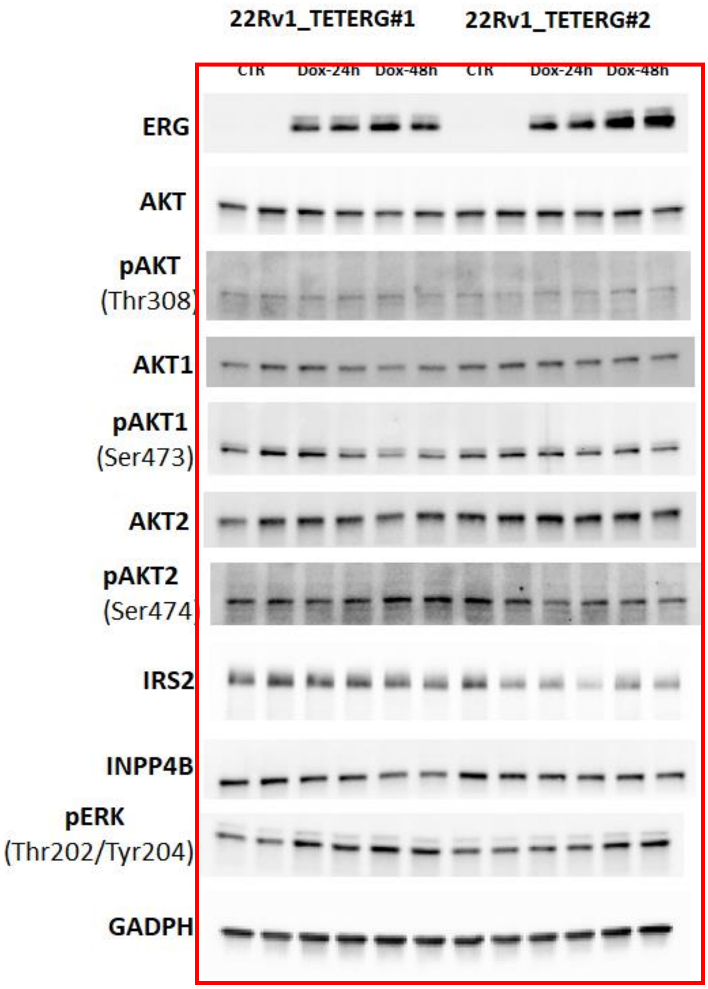

Uncropped gels corresponding to Figure S3

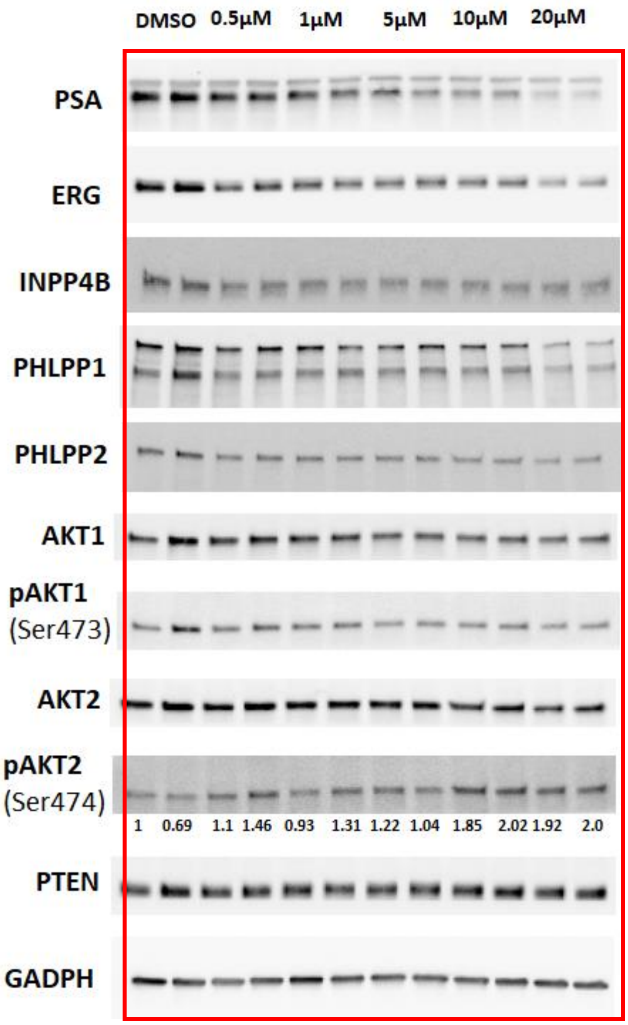

Uncropped gels corresponding to Figure S10
